# Digital Patient Decision Aids for Endometriosis Management: A Scoping Review

**DOI:** 10.1111/hex.70881

**Published:** 2026-09-25

**Authors:** Océane Pittet, Marion Delvallée, Nicola Pluchino, Kevin Selby, Glyn Elwyn, Marie‐Anne Durand

**Affiliations:** ^1^ Department of Ambulatory Care, Unisanté University Center for Primary Care and Public Health, University of Lausanne Lausanne Vaud Switzerland; ^2^ Université Claude Bernard Lyon 1, Research on Healthcare Performance RESHAPE, INSERM Lyon Rhône France; ^3^ Hospices Civils de Lyon, Service Recherche et Epidémiologie Cliniques Pôle de Santé Publique Lyon Rhône France; ^4^ Department of Gynecology and Obstetrics University Hospital of Geneva Geneva Geneva Switzerland; ^5^ The Dartmouth Institute for Health Policy & Clinical Practice, Dartmouth College Lebanon New Hampshire USA

**Keywords:** digital health, endometriosis, patient decision aid, scoping review, shared decision‐making

## Abstract

**Background:**

Endometriosis treatment requires women to navigate complex, preference‐sensitive decisions. Patient Decision Aids (PtDAs) help patients make value‐aligned choices. However, the scope and quality of digital PtDAs for endometriosis, and the cap acity of conversational AI platforms to act as PtDAs, remain unclear.

**Objectives:**

Systematically map digital PtDAs for women of reproductive age with endometriosis, describe their content, development, and evaluation, and assess quality and replicability.

**Search Strategy:**

Electronic databases, Google Scholar, grey literature, and web searches were conducted from inception to July 2025.

**Selection Criteria:**

We included digital PtDAs for women aged 18–49 with a clinical diagnosis of endometriosis that met the minimum criteria established to qualify as a PtDA. Additionally, we developed a prompt to generate five PtDAs using conversational AI platforms, mirroring patient or clinician decision‐support queries.

**Data Collection and Analysis:**

Two independent reviewers extracted data per Joanna Briggs Institute (JBI) scoping reviews methodology. IPDAS criteria (requirements for PtDAs) and TIDieR items (intervention reporting) were applied; data were summarised descriptively and qualitatively.

**Results:**

Ten PtDAs were included (five expert‐developed; five AI‐generated). Overall, most addressed pharmacological and surgical options, while AI‐generated PtDAs included more complementary therapies. All described the health condition, decision, and options with balanced pros/cons, but most failed on important IPDAS criteria for high‐quality PtDAs. Most expert‐developed PtDAs also lacked transparent development reporting and had not been formally evaluated.

**Conclusions:**

Few digital PtDAs for endometriosis were identified; most showed limited adherence to IPDAS criteria, poor reporting transparency, and absent formal evaluation.

## 1 Introduction

Endometriosis is a chronic, oestrogen‐dependent, inflammatory gynaecological disease affecting approximately 10% of reproductive age women [1, 2, 3, 4]. It is characterised by ectopic endometrial‐like tissue, leading to a wide spectrum of symptoms including chronic pelvic pain and infertility, substantially reducing quality of life [5, 6, 7, 8].

Despite increased awareness, current diagnostic and management strategies often fail to address the disease's multifaceted nature [9, 10]. Multidisciplinary care is increasingly advocated to manage physical, psychological and social health aspects [11, 12, 13]. However, no single treatment has proven universally effective, requiring women to navigate complex preference‐based choices regarding symptom relief, fertility, or both. Shared decision‐making (SDM), supported by Patient Decision Aids (PtDAs), can help women engage more effectively in this process with their healthcare providers [14]. PtDAs, as defined in the 2024 Cochrane review, are “evidence‐based tools designed to help patients make specific and deliberate choices from among healthcare options; they are intended to supplement (rather than replace) clinicians' counselling about options.” [14] PtDAs differ from usual health education materials by explicitly stating the decision to be made and providing detailed, specific, and personalised information on available options and their outcomes to support decision‐making. In contrast, health education materials aim to provide general information on diagnosis, treatment, and management, without focusing on a specific decision point, and therefore do not necessarily support active participation in decision‐making [14]. Classified into pre‐encounter and encounter categories, they can be used before, during, or after a clinical consultation, increasing patient knowledge, reducing decisional conflict, improving risk perception, and enhancing satisfaction with treatment decisions [14, 15]. Given endometriosis's complexity and lack of a universal treatment, PtDAs are particularly valuable.

Recent advances in digital technology have expanded PtDAs' potential. Web and mobile platforms offer interactive, accessible, personalised experiences, potentially improving engagement and outcomes [16] with early evidence supporting their effectiveness across clinical contexts [17, 18, 19].

Large Language Models (LLMs), delivered through conversational agents, represent a further paradigm shift: unlike static resources that require patients to synthesise fragmented information, LLMs provide context‐aware synthesis of complex evidence, enabling iterative, tailored dialogue that can simulate SDM. In this context, their widespread adoption in the general population in recent years presents novel opportunities for patient empowerment [20, 21]. Moreover, studies have shown that responses from these tools approach expert‐level accuracy regarding pathology diagnosis and treatment option [22, 23]. and recent evidence suggests LLMs may contribute to improving SDM, though their evaluation in this context remains limited [21, 22].

Despite growing interest, the landscape of digital PtDAs for endometriosis remains poorly defined. Mapping existing evidence is essential to identify current approaches, their strengths and limitations, and opportunities for future development.

The objectives of this scoping review are to systematically map digital PtDAs for women of reproductive age with endometriosis, describe their content, development, and evaluation methods, and assess quality and replicability using the International Patient Decision Aid Standards (IPDAS) and the template for intervention description and replication (TIDieR) frameworks.

## 2 Methods

This scoping review was conducted in accordance with the Joanna Briggs Institute (JBI) methodology for scoping reviews [24] and reported following the PRISMA‐ScR guidelines [25] (see Supporting Information: Appendix 1). The protocol was prospectively published [26]. As this scoping review uses data exclusively from published and publicly available sources, ethical approval was not required.

### 2.1 Search Strategy

A comprehensive three‐step search strategy was developed with an information specialist. An initial exploratory search of MEDLINE (via PubMed) and CINAHL informed the development of a full search strategy, which was then adapted for each database. The complete search strategies for all databases are provided in Supporting Information: Appendix 2.

**Figure 1:**
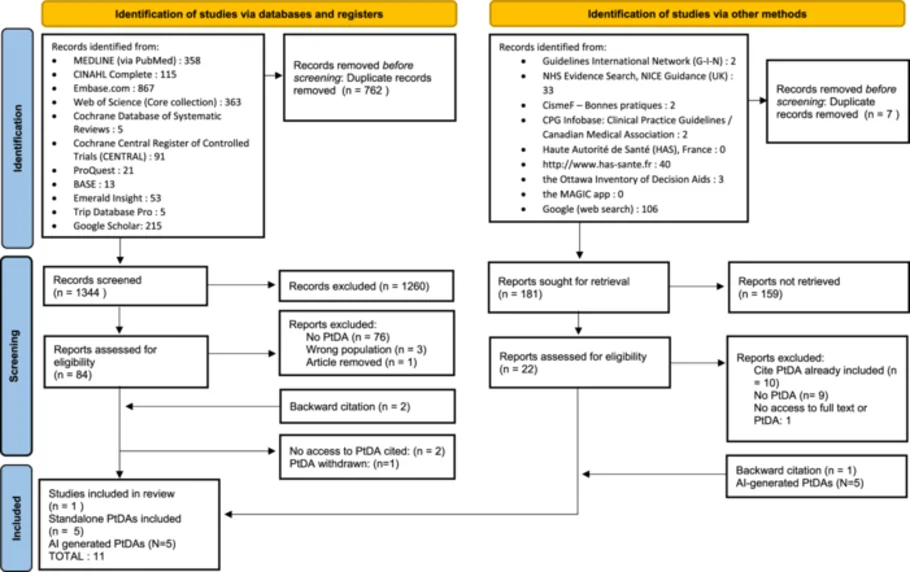
PRISMA flow diagram.

Ten electronic databases or registers as well as Google Scholar were searched from inception to July 2025 supplemented by grey literature and Google searches. The databases and registers included in the search are listed in Figure 1.

### 2.2 Eligibility Criteria and Study Selection

The review was guided by the following question: What are the characteristics, development processes, and evaluation methods of digital PtDAs designed for women of reproductive age with endometriosis? The objectives were to (1) identify and describe the content and features of existing digital PtDAs; (2) examine their development processes, including stakeholder involvement; and (3) summarise reported evaluation methods and outcomes.

Eligibility criteria followed the Population–Concept–Context framework [27]. We included studies and resources involving women aged 18–49 years with a clinical diagnosis of endometriosis. Eligible records needed to describe, develop, implement, or evaluate a PtDA meeting the minimal IPDAS definition, namely: clearly stating the decision to be made, presenting evidence‐based information on options and their benefits and harms, and helping patients clarify the values they place on outcomes [28]. Eligible tools were purpose‐built digital PtDAs remotely accessible via web, mobile, or personal digital technologies.

Given patients' growing use of AI to seek health information and support decision‐making, we distinguished between human‐led and AI‐generated PtDAs.

All study designs were eligible, including grey literature and standalone PtDAs. We excluded PtDAs outside the target population, non‐digital resources, materials not meeting minimal PtDA criteria, and inaccessible records.

Citations were imported into Rayyan [29] and deduplicated. Two independent reviewers (OP and MD) screened titles, abstracts, and full texts; disagreements were resolved by discussion or third‐reviewer consultation. Authors were contacted when eligibility was uncertain or PtDAs were inaccessible; records were excluded if no response was received.

To map the emerging role of generative AI in decision support and mirror the experience of a patient or clinician seeking treatment decision support for endometriosis, we examined AI‐generated outputs by creating five digital PtDAs using major conversational AI platforms. For the platform‐selection stage, we focused on established, consumer‐facing generative AI tools with broad adoption. As the identified platforms were subsequently used to generate health‐related content, data security and safety considerations were also taken into account when determining their suitability for evaluation. These considerations were applied during the conduct of the review and were not prespecified as eligibility criteria in the published protocol. From these, we selected the most widely used platforms worldwide, namely OpenAI ChatGPT (GPT‐4o), Anthropic Claude (Claude Sonnet 4.5), Google Gemini (Gemini 2.5 Flash), Microsoft Copilot (standard non‐premium configuration), and Perplexity AI (standard non‐premium configuration) [30, 31].

For Microsoft Copilot and Perplexity AI, the specific underlying model could not be independently verified and was therefore not attributed to a single model.

Model selection followed a consistent criterion across platforms: we used the model or configuration provided by default under free access conditions; platforms were accessed anonymously where account‐free access was available or through dedicated free, non‐premium accounts where sign‐in was required. We chose this approach rather than matching computational tier across vendors, since tier classifications are not standardised or comparable across providers. To further standardise conditions across platforms and minimise potential personalisation bias. Together, these procedures were intended to approximate the experience of a first‐time general user accessing each platform under standard, freely available conditions. Each platform received an identical, intentionally brief prompt (“*Generate a patient decision aid for individuals with endometriosis”*) with no follow‐up instructions, to reflect a typical real‐world user query and assess baseline unassisted performance. All generations were performed on 17 October 2025. The specific technical configurations and raw AI‐generated PtDA outputs are documented in Supporting Information: Appendix 3. This component was conducted for exploratory purposes to assess the feasibility and characteristics of PtDAs generated by current generative AI systems, rather than as a definitive benchmarking or comparative evaluation against established PtDAs. The screening and selection process is summarised in a PRISMA flow diagram (Figure 1).

### 2.3 Data Extraction and Synthesis

Two reviewers (OP and MD) independently extracted data using a standardised, pilot‐tested charting form adapted from the JBI template, covering bibliographic and methodological characteristics, study population and setting, PtDA definition, digital components, content and features, development processes, evaluation methods, patient involvement, and outcomes (See Supporting Information: Appendices 4 and 5 for the data extraction materials.) IPDAS qualifying and certification criteria [32] were applied to assess quality and replicability, excluding four certification criteria specific to tests or screening. TIDieR checklist items [33] were used to assess intervention replicability, excluding the “WHERE” item as digital delivery was an inclusion criterion. TIDieR was used to assess the completeness of intervention descriptions, rather than to evaluate their clinical or intrinsic quality or whether they were appropriately designed for their intended purpose. Details of applied IPDAS criteria and TIDieR items are provided in Supporting Information: Table S1.

AI‐generated PtDAs were extracted using the same data extraction sheet to ensure consistency and enable comparison with PtDAs identified in the literature.

Extracted data were collated and summarised using descriptive statistics and qualitative content analysis to identify key characteristics, recurring themes, and gaps in the literature. Summary tables and figures are presented in the Results section.

### 2.4 Patient and Public Involvement

Two patient partners were integrated into the research team. Their contributions are summarised in Table 1 below according to the GRIPP2‐SF checklist [34].

**Table 1:** Patient and public involvement in the study reported according to the GRIPP2‐SF checklist [34].

Aim	Ensure that the research project and manuscript accurately reflected the perspectives and priorities of individuals living with endometriosis.
Methods	Two patient partners were integrated into the research team. They participated in discussions on project development and reviewed draft manuscripts. Documents were translated into the patient partners' native language, and additional explanations were provided for methodological concepts as needed. Patient partners received financial compensation for their contributions.
Results	Patient partners contributed to shaping the project and refining the manuscript, ensuring that outputs were understandable, relevant, and aligned with patient perspectives.
Discussion and conclusions	Involvement of patient partners strengthened the project and manuscript by grounding them in real‐world experience. While their engagement was limited to specific tasks, structured communication and clear materials facilitated meaningful contributions.
Reflections/critical perspective	Challenges included coordinating availability with research timelines and explaining methodological details. Providing clear, accessible documents and maintaining open communication were key to successful engagement.

## 3 Results

### 3.1 Study Inclusion

Database searches yielded 2106 records of which 1344 remained after deduplication. Grey literature and targeted Google searches added 82 and 106 records respectively, bringing the overall number of records screened to 1525 after removing 7 duplicates.

Following title and abstract screening, 106 records underwent full‐text assessment (84 from databases, 22 from grey literature), of which ten met inclusion criteria, corresponding to nine unique PtDAs.

Four records were excluded due to inaccessible PtDAs: three authors did not respond to contact attempts, and one confirmed their PtDA had been withdrawn following a 2024 guideline update. The list of records excluded due to inaccessibility is provided in Supporting Information: Appendix 6. The Five PtDAs were retained for data extraction: three in English, one in Spanish/Catalan, and one in Dutch; non‐English PtDAs were translated using automatic browser translation. These were identified through a conference abstract backward citation (*n* = 2) and direct Google searches (*n* = 3).

Five additional PtDAs were generated using major conversational AI platforms (ChatGPT, Gemini, Claude, Microsoft Copilot, Perplexity). All met inclusion criteria and were included in the analysis.

The study selection process is summarised in the PRISMA flow diagram (Figure 1).

### 3.2 General Characteristics of Included PtDAs

For clarity, each PtDA was assigned a short identifier based on its country and developer: ES–Gencat [35, 36], US–Healthwise [37], DE–IQWiG [38], UK–StGeorge [39], NL–UMC [40], and AI‐generated PtDAs (AI–ChatGPT, AI–Perplexity, AI–Claude, AI–Microsoft Copilot, AI–Gemini).

Five PtDAs developed by health experts met the inclusion criteria [35, 36, 37, 38, 39, 40]. Characteristics of PtDAs are provided in Table 2. Their creation or most recent update dates ranged from 2018 to 2025 and they originated from five countries: Spain, the United Kingdom, the Netherlands, Germany, and the United States. All targeted women with endometriosis and were developed to support treatment decisions, including medical management, surgical interventions, and fertility preservation. All were pre‐encounter PtDAs.

**Table 2:** Characteristics of included PtDAs.

Abbreviation	Country (year of creation or last update/generation	Developer	Medical treatment options covered	Surgical treatment options covered	Complementary or alternative options covered	Fertility options covered
ES–Gencat [35, 36]	Spain (2024)	Catalan Ministry of Health	NSAIDs, opioids, antiepileptics and antidepressants (neuropathic pain); hormonal treatment: progestogens, oestrogens and GnRH	Laparoscopy, Hysterectomy, Oophorectomy and Cystectomy	Dietary, physical therapy, psychotherapy, acupuncture	Assisted reproduction (with or without IVF), surgery, oocyte and embryo preservation
US–Healthwise [37]	United States (2025)	WebMD Ignite Healthwise	NA	Laparoscopy, Hysterectomy and Oophorectomy	NA	NA
DE–IQWiG [38]	Germany (2024)	IQWiG	NSAIDs and hormonal treatments: progestogens, contraceptive pills, GnRH (agonists and antagonists)	Laparoscopy and hysterectomy	NA	NA
UK–StGeorge [39]	United Kingdom (2024)	St George's University Hospitals NHS Foundation Trust	NSAIDs and hormonal treatments: progestogens, contraceptive pills and GnRH antagonists	Laparoscopy and hysterectomy	NA	NA
NL–UMC [40]	Netherlands (2018)	Amsterdam UMC	NA	NA	NA	Oocyte and embryo preservation

Abbreviation	Country (Year of creation or last update/generation	Platform name (underlying model)	Medical treatment options covered	Surgical treatment options covered	Complementary or alternative options covered	Fertility options covered
AI–ChatGPT	USA (2025)	OpenAI ChatGPT (GPT‐4o)	NSAIDs and hormonal treatments: contraceptive pills, GnRH agonists and IUD	Laparoscopy and hysterectomy	Dietary, acupuncture, physical therapy	IVF
AI–Perplexity	USA (2025)	Perplexity AI (standard non‐premium configuration)	Paracetamol, NSAIDs, hormonal treatments: contraceptive pills, progestogens, oestrogens and GnRH	Laparoscopy, hysterectomy and Oophorectomy	Dietary, physical therapy, acupuncture	Surgery
AI–Claude	USA (2025)	Anthropic Claude (Claude Sonnet 4.5)	Painkillers (not specified), hormonal treatment: contraceptive pills, IUD and others (not clearly specified)	Laparoscopy, hysterectomy and oophorectomy	Dietary, physical therapy, acupuncture and stress management	NA
AI–Microsoft Copilot	USA (2025)	Microsoft Copilot (standard non‐premium configuration)	NSAIDs, hormonal treatment: progestogens, contraceptive and GnRH (agonist)	Laparoscopy, hysterectomy and oophorectomy	Dietary, physical therapy, acupuncture and stress management, exercise	NA
AI–Gemini	USA (2025)	Google Gemini (Gemini 2.5 Flash)	NSAIDs, hormonal treatment: progestogens, contraceptive and GnRH (agonist or antagonists), IUD, Patch and Ring	Laparoscopy, hysterectomy and oophorectomy	NA	NA

Three were web‐based interactive platforms; two were downloadable PDFs. All but one (US–Healthwise) were developed by public or academic institutions. Only one PtDA (ES‐Gencat) had an associated conference abstract describing its co‐creation and preliminary evaluation as well as usage statistics and user satisfaction. For all other PtDAs, development information was limited to the tool itself, and no evaluation data were available.

We generated five PtDAs on 17 October 2025 using AI‐platforms operated by US‐based companies. All were pre‐encounter PtDAs, except Gemini's output which incorporated encounter‐type elements. Notably, Claude automatically produced two outputs, one featuring tables and colour highlighting.

### 3.3 Content of PtDAs

#### 3.3.1 PtDAs Developed by Health Experts

All five expert‐developed PtDAs addressed pharmacological treatments. ES–Gencat, DE–IQWiG, and UK–St George covered NSAIDs, hormonal therapies, and pain management; ES–Gencat additionally addressed neuropathic pain. Surgical options were covered in four PtDAs, while US–Healthwise focused specifically on hysterectomy versus oophorectomy. Fertility and reproductive options were addressed in ES–Gencat, DE–IQWiG, and NL–UMC, the latter being exclusively dedicated to fertility preservation. Only ES–Gencat considered broader quality of life aspects by addressing lifestyle and complementary interventions. Full details on PtDA content are provided in Table 2.

Decision‐support features, including value‐clarification exercise, treatment comparison tools and consultation preparation support, were incorporated in three PtDAs (ES–Gencat, US–Healthwise, and DE–IQWiG). In contrast, UK–St George and NL–UMC's contained no explicit decision‐support features. ES–Gencat and DE–IQWiG also included patient testimonials, either written or in video format.

#### 3.3.2 AI‐Generated Ptdas

All five AI‐generated PtDAs covered pharmacological management and surgical interventions. All except AI‐Gemini also included complementary therapies. Fertility preservation and assisted reproduction options were explicitly addressed in one PtDA (AI‐Gemini), though all noted the potential impact of endometriosis treatments on fertility. Full details on PtDA content are provided in Table 2.

All included decision‐support sections with prompts on key decision factors and questions for healthcare professionals; all except AI‐Perplexity incorporated explicit values clarification. AI‐ChatGPT, AI‐Claude, and AI‐Gemini outlined next steps in the decision process.

#### 3.3.3 All PtDAs

Across all 10 PtDAs, nine addressed users in the first‐person (e.g., “I”) whereas one (Perplexity AI) used a third‐person, descriptive format. All defined the SDM process and emphasised the importance of discussing the information with a healthcare professional.

None explicitly addressed endometriosis staging or classification but several noted that treatment decisions may depend on symptom severity or type of endometriosis and emphasised the importance of discussing these factors with a healthcare professional.

Most PtDAs (9/10) included an overview or definition of endometriosis, except for NL‐UMC which omitted this, focusing solely on fertility preservation. All presented treatment options with advantages and disadvantages.

### 3.4 Development of PtDAs

Among expert‐developed PtDAs, reporting on development processes was limited, and patient and stakeholder involvement varied considerably. ES‐Gencat reported a co‐creation process including stakeholders, healthcare institutions, clinicians, patients and patient associations. US‐Healthwise was developed by a multidisciplinary team (clinicians, writers, editors and external contractors) but did not describe patient involvement or detail the development process. NL‐UMC was informed by eight brainstorming sessions with clinicians, a psychologist and patient experts. DE‐IQWiG involved researchers, clinicians and journalists drawing on research evidence and patient needs, though the process for integrating patient input was not described. UK–St George provided no development information.

AI‐generated PtDAs were created entirely in response to a prompt, with no information on development processes, content sourcing, or methodology.

### 3.5 IPDAS Criteria

The criteria used to assess whether each IPDAS criterion was met were defined by the study authors and are detailed in Supporting Information: Table S2. Detailed results for each PtDA against the IPDAS qualifying and certification criteria are presented in Table 3.

**Table 3:** Assessment of IPDAS criteria fulfilment across included tools.

Criteria	ES–Gencat	US–Healthwise	DE–IQWiG	UK–StGeorge	NL–UMC	AI‐ChatGPT	AI‐Gemini	AI‐Claude	AI‐Microsoft Copilot	AI‐Perplexity
Description of health condition	✓	✓	✓	✓	✓	✓	✓	✓	✓	✓
States the decision to be considered	✓	✓	✓	✓	✓	✓	✓	✓	✓	✓
Describes the options available	✓	✓	✓	✓	✓	✓	✓	✓	✓	✓
Positive features of options	✓	✓	✓	✓	✓	✓	✓	✓	✓	✕
Negative features of options	✓	✓	✓	✓	✓	✓	✓	✓	✓	✕
Experience of consequences	✓	✓	✕	✕	✕	✕	✕	✕	✕	✕
Equal detail for pros/cons	✓	✓	✓	✕	✕	✓	✓	✓	✓	✕
Citations to evidence	✓	✕	✕	✕	✕	✕	✕	✕	✕	✓
Production/publication date	✓	✓	✓	✓	✓	✕	✕	✕	✕	✕
Update policy	✕	✓	✓	✕	✕	✕	✕	✕	✕	✕
Levels of uncertainty	✓	✕	✓	✓	✕	✕	✕	✕	✕	✕
Funding source	✕	✓	✓	✕	✕	✕	✕	✕	✕	✕

#### 3.5.1 PtDAs Developed by Health Experts

All expert‐developed PtDAs described the health condition, stated the decision to be made, reported a production date and described available options with pros and cons, though two provided unequal detail across options (UK‐StGeorge and NL‐UMC). Experience of consequences were addressed in two PtDAs (ES‐Gencat and US–Healthwise), through personal stories and FAQs. One (ES‐Gencat) provided a bibliography documenting the sources used to develop its clinical content; others cited their development team, search methods, or format‐related references. Two (US–Healthwise and DE‐IQWIG) specified their update policy via a link embedded in the PtDA directing users to a webpage. Uncertainty around outcome probabilities was quantified in three PtDAs (ES‐Gencat, DE‐IQWiG, UK‐StGeorge); the two others expressed it in qualitative terms only. Two PtDAs (US–Healthwise and DE‐IQWIG) disclosed funding sources.

#### 3.5.2 AI‐Generated PtDAs

All five AI‐generated PtDAs described the health condition, stated the decision to be made, and described available options with pros and cons; all but one (AI‐Perplexity) presented these options with comparable detail. Only AI‐Perplexity cited supporting evidence. None described experiential consequences, reported outcome uncertainty surrounding outcomes, provided update policies, production dates, or funding sources.

### 3.6 TiDieR Checklist

Brief names were derived from PtDA titles, and delivery mode corresponded to eligibility criteria (digital PtDAs accessible remotely via web‐based platforms, mobile applications, or other personal digital technologies). All PtDAs targeted women with endometriosis but none reported individual tailoring. Insufficient information precluded extraction of other TIDieR items; the only potentially relevant report was a conference abstract [35], but the information provided remained too limited to support a structured TIDieR‐based analysis.

## 4 Discussion

### 4.1 Main Findings

This scoping review included 10 PtDAs supporting shared decision‐making in endometriosis: five developed by health experts and five generated by conversational AI. Most focused on pharmacological and surgical interventions with limited coverage of complementary and alternative therapies. Both expert‐ and AI‐developed PtDAs addressed core IPDAS criteria yet important gaps were identified: Experiential consequences, evidence citations, uncertainty reporting, update policies, and funding sources were frequently missing. Only one PtDA (ES‐Gencat) has been reported in the scientific literature, but the information describing the PtDA's development and its effect remains sparse. Reporting was limited, precluding a comprehensive TIDieR‐based assessment. Only basic information, such as the PtDA title, mode of delivery and tailoring, could be extracted, while details on other intervention characteristics were largely absent or insufficiently reported. Nevertheless, some PtDAs provided brief descriptions of their development process, offering partial insight into their design rationale.

### 4.2 Interpretation

A key finding is the minimal coverage of complementary and alternative therapies in available expert‐generated digital PtDAs for endometriosis, likely reflecting reliance on clinical guidelines that seldom endorse such approaches. The NICE Guideline [41] for instance, does not support traditional Chinese medicine or lifestyle interventions due to insufficient evidence. Yet these approaches are widely used: the ESHRE Guideline cites Schwartz et al. [42] reporting that 62.5% of patients in Switzerland, Austria, and Germany use non‐medical strategies, often driven by dissatisfaction with conventional care, and Armour et al. [43] found that over 70% of individuals with endometriosis use self‐management techniques such as heat, dietary changes, meditation, or breathing exercises. These practices often reflect patients' desire to regain control over their condition [44]. The updated ESHRE [11] and RANZCOG [45] guidelines have begun integrating emerging evidence on acupuncture, physiotherapy, psychological interventions, and nutrition, while advising caution regarding cost and limited evidence base. In contrast, most AI‐generated PtDAs included complementary or lifestyle‐based approaches, suggesting a broader scope of information not strictly limited to clinical guideline recommendations. This difference may reflect the fact that AI systems draw on diverse publicly available sources rather than being constrained by formal guideline frameworks.

A recent NHS PtDA for chronic primary pain [46] illustrates how non‐medical options can be effectively integrated into PtDAs using a hierarchical architecture, organising 15 options under two overarching categories. It is a model well‐suited to endometriosis, where patients must balance hormonal treatments, pain management, and lifestyle adjustments. Notably, in our exploratory development of PtDAs using conversational AI platforms, four out of five AI‐generated prototypes spontaneously included complementary therapies, underscoring the perceived relevance of such options. However, it is essential that PtDAs clearly inform patients when evidence is scarce or inconclusive, particularly concerning treatment efficacy, adverse effects, and potential interactions with other therapies. These findings highlight the importance of co‐developing PtDAs with patients and clinicians to reflect both evidence and lived experience.

Regarding accessibility, Google searches conducted via a neutral account retrieved some of the identified PtDAs, whereas searches performed via personal accounts (previously used to explore topics related to endometriosis and patient decision aids), yielded additional results. This suggests that algorithmic personalisation may limit equitable access to PtDAs for patients with limited prior engagement with endometriosis content.

In relation to our review question and objectives, it is noteworthy that the available evidence on content, development, and evaluation methods is extremely limited. Only a single publication was retrieved (conference abstract with minimal detail) and no full‐text articles reported on the development, evaluation, or outcomes of digital PtDAs for endometriosis. While decision aids have been widely developed in other clinical areas, endometriosis represents a uniquely preference‐sensitive condition, as no treatment has consistently demonstrated clinical superiority, making shared decision‐making and high‐quality PtDAs particularly critical. Consequently, patients must navigate uncertain and often complex therapeutic trajectories, shaped by pain management, fertility considerations, treatment burden, and long‐term quality‐of‐life outcomes. These characteristics make high‐quality patient decision aids particularly relevant in this field. Data evaluating effectiveness against established outcomes, including concordance with patient values, knowledge acquisition, or decisional quality, are largely absent. Key quality determinants such as rigour of evidence synthesis and transparency regarding competing interests are rarely reported. Importantly, while transparency gaps are especially pronounced for AI‐generated PtDAs, they are broadly shared: most expert‐developed PtDAs equally failed to meet IPDAS criteria related to evidence citation, update policies, and funding disclosure. Overall, the lack of detailed reporting regarding design processes, stakeholder involvement, and assessment procedures substantially limits appraisal, reproducibility, and comparability of future research in this area.

AI‐generated PtDAs face additional structural constraints: as LLMs generate content dynamically from prompts rather than through a structured development process and lived experience, they are inherently limited in addressing experiential consequences of treatment options. Furthermore, depending on the system design, they may not consistently provide verifiable and traceable citations, as outputs are synthesised probabilistically from vast datasets rather than linked to traceable sources [47, 48, 49].

Finally, the rapid evolution of digital and AI‐enabled PtDAs raises questions about the ongoing applicability of current IPDAS criteria, which were primarily developed for static formats. As digital PtDAs increasingly incorporate interactive, adaptive, and generative features, existing standards may need to be revisited to remain fit for purpose.

### 4.3 Strength and Limitation

Strengths include a comprehensive search across multiple databases, grey literature, and targeted web searches in English and French, supported by rigorous methodological frameworks (JBI, PRISMA‐ScR, IPDAS, TIDieR). The inclusion of AI‐generated PtDAs provided novel insights into emerging technologies. Limitations include the absence of formal IPDAS training, which may have introduced variability. In addition, The Cochrane and IPDAS definitions of PtDAs rely on specific criteria that may exclude informational, educational, or supportive interventions that do not formally qualify as PtDAs. Consequently, some resources that may meaningfully assist patients in making healthcare decisions may not be captured within this classification, potentially limiting the breadth of interventions considered in this review. Moreover, limited methodological detail in most PtDAs constrained the evaluation of replicability, some PtDAs may have been inaccessible, and findings from AI‐generated PtDAs should be interpreted with caution as they have not yet been evaluated in real‐world settings. In addition, the included generative AI platforms are all US‐based, reflecting those that currently meet our predefined criteria for adoption and verified data security standards, which may limit generalisability.

Furthermore, the AI‐generated PtDAs were produced using a single, simple, and intentionally exploratory prompt, without iterative refinement or follow‐up queries. This choice was made to approximate a realistic first‐user interaction with generative AI systems, rather than to implement an optimised or standardised prompt engineering strategy. The approach was informed by input from a multidisciplinary steering committee, including clinicians and patient partners. Importantly, there is currently no established evidence base or validated framework for optimal prompt design in the generation of PtDAs using generative. We therefore acknowledge that real‐world performance may differ depending on user intent, expertise, and prompting strategies, and that future research is needed to develop and evaluate systematic approaches to prompt design in this context.

## 5 Conclusions

Despite growing recognition of the importance of shared decision‐making in endometriosis care, digital PtDAs remain scarce, poorly evaluated, and lack transparency regarding key IPDAS criterias. AI‐generated PtDAs covered a broader choice of treatment options but lacked key elements. There is a clear need for co‐created, accessible, and comprehensive PtDAs that integrate medical, and self‐management options, while providing transparent information about its development. Future research should rigorously report the development of PtDAs and evaluate their effectiveness. Furthermore, the evolving role of artificial intelligence in generating, personalising, and scaling PtDAs warrants systematic investigation to ensure safety, reliability, and clinical integration.

## Author Contributions

**Océane Pittet:** conceptualisation, methodology, formal analysis, writing – original draft, writing – review and editing, investigation, visualisation, funding acquisition, project administration, validation. **Marion Delvallée:** conceptualisation, methodology, formal analysis, writing – original draft, writing – review and editing, investigation, validation. **Nicola Pluchino:** conceptualisation, methodology, writing – review and editing, funding acquisition. **Kevin Selby:** conceptualisation, methodology, writing – review and editing. **Glyn Elwyn:** conceptualisation, methodology, supervision, writing – review and editing. **Marie‐Anne Durand:** conceptualisation, methodology, writing – review and editing, supervision, investigation, validation, funding acquisition, project administration, formal analysis, visualisation.

## Disclosure

In this manuscript, the term ‘women’ refers to individuals born with a uterus. We recognise that not all people with a uterus at birth identify as women. Generative AI tools were used for language refinement and editing to improve the clarity, grammar, and readability of the manuscript. All final decisions regarding the content of the manuscript were made by the authors. Lived Experience or Public Contribution: Two patient partners with lived experience of endometriosis were involved in project development and manuscript review. Materials were translated and methodological concepts clarified to support engagement, and partners were compensated for their contributions. Their input improved the relevance, clarity, and accessibility of the study.

## Conflicts of Interest

M.A.D. and G.E. have contributed to the development of Option Grid patient decision aids. EBSCO Information Services sells subscription access to Option Grid patient decision aids. They receive consulting income from EBSCO Health. No other competing interests declared. The other authors declare no conflicts of interest.

## Supporting information


Supporting File 1

Supporting File 2

Supporting File 3

Supporting File 4

Supporting File 5

Supporting File 6

Supporting File 7

Supporting File 8

## Data Availability

The data that supports the findings of this study are available in the supplementary material of this article.
