# Supplementary material for: Digital Patient Decision Aids for Endometriosis Management: A Scoping Review
Source: Health Expect. 2026 Sep 25;29(5):e70881. doi: 10.1111/hex.70881 (PMC13614076; doi:10.1111/hex.70881)
Supplement: Supplementary file 1 [file HEX-29-e70881-s001.docx]

# Appendix 3

All simulations were conducted using the standard, non-premium configuration available on each respective platform at the time of data collection. Platforms were accessed anonymously where account-free access was available or through dedicated free, non-premium accounts where sign-in was required.

Internet access was systematically enabled for all platforms; however, advanced specialized features, such as deep-research modes or reasoning-specific modes, were disabled.

The same standardized prompt was submitted to each platform without modification: “Generate a patient decision aid for individuals with endometriosis.”

The platforms and model configurations used were: OpenAI ChatGPT (GPT-4o), Anthropic Claude (Claude Sonnet 4.5), Google Gemini (Gemini 2.5 Flash), Microsoft Copilot (standard non-premium configuration), and Perplexity AI (standard non-premium configuration). For Microsoft Copilot and Perplexity AI, which may dynamically route queries through different underlying models, the specific underlying model could not be independently verified and is therefore not attributed to a single model.

No attempt was made to select models of equivalent performance tiers across providers. Rather, the configurations accessible under the study conditions were used to reflect their standard use at the time of data collection. Accordingly, the use of Claude Sonnet 4.5 and Gemini 2.5 Flash reflects the configurations available on the respective platforms rather than a deliberate selection of Sonnet and Flash as comparable model tiers.

All generations were performed on October 17, 2025.

# OpenAI ChatGPT

Here is a Patient Decision Aid (PDA) designed for individuals with endometriosis, helping them understand their condition, explore treatment options, weigh benefits and risks, and make informed choices in collaboration with their healthcare team.

**🌸 Endometriosis: Patient Decision Aid**

For people diagnosed with or suspected to have endometriosis

✅ What Is This Tool?

This Patient Decision Aid helps you:

- Understand what endometriosis is
- Learn about your treatment options
- Consider what's important to *you*
- Prepare for a conversation with your doctor

1. 🤔 What Is Endometriosis?

Endometriosis is a condition where tissue similar to the lining of the uterus grows outside the uterus. This can cause:

- Chronic pelvic pain
- Pain during menstruation or sex
- Heavy periods
- Fatigue
- Infertility

It's a chronic condition, but it can be managed with the right treatment plan.

2. 🩺 What Are My Treatment Options?

| Option | Pros | Cons | Suitable for |
| --- | --- | --- | --- |
| Watchful Waiting | No medication side effects; suitable for mild symptoms | Symptoms may worsen or persist | Mild/no pain; not trying to conceive |
| Pain Relief (NSAIDs) | Easy to access; helps with cramps and inflammation | Does not treat the root cause | Mild to moderate symptoms |
| Hormonal Therapy (e.g. birth control pills, IUD, GnRH agonists) | Can reduce or stop periods; slows lesion growth | Side effects (mood changes, bone loss, hot flashes); not suitable when trying to conceive | Moderate to severe symptoms; not planning pregnancy |
| Surgery (laparoscopy) | Removes lesions; may improve fertility | Invasive; risk of recurrence; recovery time | Severe symptoms; infertility; when meds don’t help |
| Fertility Treatment (e.g. IVF) | Helps with conception | Expensive; physically and emotionally demanding | Trying to get pregnant |
| Complementary Therapies (diet, acupuncture, physical therapy) | Holistic; non-invasive | Evidence varies; may not work for all | Those preferring natural or supportive care |
| Hysterectomy (removal of uterus ± ovaries) | Can reduce symptoms long-term | Permanent; not a cure; ends fertility | Completed family; severe symptoms unresponsive to other treatments |

3. 🧠 Things to Think About

Ask yourself:

- How much are my symptoms affecting my daily life?
- Do I want to have children now or in the future?
- How do I feel about taking hormones or having surgery?
- Do I prefer natural or medical treatments?
- What are my hopes or fears about treatment?

4. 🧭 What Matters Most to You?

| What’s Important to Me | ✔️ |
| --- | --- |
| I want to get relief from pain |  |
| I want to get pregnant soon |  |
| I want to avoid surgery |  |
| I want the most effective treatment |  |
| I want to avoid hormone-based medications |  |
| I want a reversible treatment option |  |
| I prefer natural or complementary approaches |  |

(*Check the boxes that apply to you*)

5. 💬 Questions to Ask Your Healthcare Provider

- What type of endometriosis do I have?
- What are the benefits and risks of each treatment for me?
- How will treatment affect my fertility?
- What are my non-surgical options?
- What happens if I do nothing for now?
- What are the chances of recurrence?

6. 📝 My Next Steps

☐ Talk to my doctor about treatment options
☐ Track my symptoms for the next few weeks
☐ Get a second opinion (if needed)
☐ Explore support groups or counseling
☐ Make a treatment decision by: ______________

7. 🧍‍♀️ Support & Resources

- Endometriosis Foundation of America: [https://www.endofound.org](https://www.endofound.org/)
- Endometriosis UK: [https://www.endometriosis-uk.org](https://www.endometriosis-uk.org/)
- MyEndometriosisTeam: Online support group
- Pelvic pain specialists & physical therapists

8. 📌 Summary

There is no “one size fits all” solution for endometriosis. The best choice depends on your:

- Symptoms
- Fertility goals
- Preferences
- Lifestyle

This is your body, your life, and your choice. Use this tool to guide conversations with your care team.

# Perplexity AI

A patient decision aid for individuals with endometriosis is designed to support informed, shared decision-making by providing clear, balanced information about treatment options, benefits, risks, and the impact on quality of life. The aid should help patients clarify their values and preferences, empower self-advocacy, and prepare them for discussions with their healthcare providers.

Key components of such a decision aid include:

Understanding Endometriosis

- Endometriosis is a chronic condition where tissue similar to the uterine lining grows outside the uterus, causing pain and possibly affecting fertility.
- There is no cure, but symptoms can be managed with various treatments.

Treatment Options

1. Medications:
   - Pain relief (e.g., paracetamol, ibuprofen)
   - Hormonal therapies to reduce or stop menstruation and slow endometriosis tissue growth:
     - Combined hormonal contraceptives (pills, patches, rings)
     - Progestin therapies (pill, hormonal IUD, injections)
     - Gonadotropin-releasing hormone (GnRH) agonists and antagonists (may induce an artificial menopause)
     - Aromatase inhibitors (reduce estrogen levels) often combined with other hormonal treatments
   - Note: Hormone treatments do not cure endometriosis; symptoms may return after stopping medication.
2. Surgical Options:
   - Laparoscopy to remove or destroy endometriosis lesions or cysts (endometriomas)
   - More extensive surgery (e.g., hysterectomy, ovary removal) in severe cases or when fertility is not desired
   - Surgery may improve fertility in mild cases but involves risks of recurrence and complications.
3. Alternative and Complementary Therapies:
   - Some patients find relief through acupuncture, dietary changes, or physical therapy, but evidence varies.
4. Choosing Not to Treat:
   - For mild symptoms, watchful waiting and symptom management may be appropriate.

Considerations for Decision-Making

- Impact on fertility goals: Hormone therapies typically prevent pregnancy; surgery may have fertility risks or benefits.
- Side effects and impact on quality of life, including emotional and mental health aspects.
- Financial costs and access to treatments.
- Balancing treatment effectiveness with personal values and family considerations.
- The emotional toll and need for ongoing support.

How to Use This Aid

- Review each treatment option’s pros and cons.
- Reflect on personal goals, lifestyle, and preferences.
- Discuss questions and concerns with healthcare providers.
- Consider involving family or support persons in discussions.
- Use the aid repeatedly as symptoms and circumstances change.

Additional Resources

- Educational materials (pamphlets, videos)
- Support groups and counseling for emotional support

This decision aid is intended to facilitate shared decision-making between patients and healthcare professionals, helping individuals with endometriosis make choices aligned with their values and life goals. It recognizes that endometriosis management is highly individualized, and ongoing reassessment is important.

# Anthropic Claude

Endometriosis Patient Decision Aid

Making informed choices about your treatment options

Understanding Endometriosis

Endometriosis is a chronic condition where tissue similar to the lining of the uterus grows outside the uterus. This can cause pain, heavy periods, and may affect fertility. It affects approximately 1 in 10 women of reproductive age.

Common Symptoms

Painful periods, chronic pelvic pain, pain during intercourse, painful bowel movements or urination (especially during periods), heavy menstrual bleeding, fatigue, and difficulty getting pregnant.

Your Treatment Options

There is no cure for endometriosis, but various treatments can help manage symptoms. The best choice depends on your symptoms, goals, and preferences.

1. Pain Management (Watchful Waiting)

For mild symptoms, you may choose to manage pain with over-the-counter medications and lifestyle changes without medical intervention.

Benefits

- No side effects from medications
- Non-invasive approach
- Low cost
- Maintains fertility

Risks/Limitations

- May not adequately control pain
- Endometriosis may progress
- Symptoms may worsen over time
- May affect quality of life

2. Hormonal Therapy

Birth control pills, hormonal IUDs, or other hormonal medications can reduce or eliminate menstruation, helping to control endometriosis growth and pain.

Benefits

- Can significantly reduce pain
- Non-surgical option
- Can regulate or stop periods
- Reversible treatment

Risks/Limitations

- Side effects (mood changes, weight gain)
- Not suitable if trying to conceive
- Symptoms may return after stopping
- Requires ongoing medication

3. Laparoscopic Surgery (Excision or Ablation)

Minimally invasive surgery to remove or destroy endometriosis tissue. This is the only way to definitively diagnose and treat endometriosis simultaneously.

Benefits

- Can provide long-term pain relief
- May improve fertility
- Confirms diagnosis
- Can be effective for severe cases

Risks/Limitations

- Surgical risks (bleeding, infection)
- Recovery time needed
- Endometriosis may recur
- More invasive than medications

4. Hysterectomy (with or without removing ovaries)

Surgical removal of the uterus, and sometimes the ovaries. This is typically considered when other treatments have failed and you don't wish to have children in the future.

Benefits

- May provide definitive relief
- No more menstrual periods
- Can improve quality of life
- Eliminates pregnancy concerns

Risks/Limitations

- Permanent loss of fertility
- Major surgery with risks
- May cause early menopause
- Endometriosis can still persist

5. Complementary Therapies

Physical therapy, acupuncture, dietary changes, and stress management can be used alongside medical treatments to help manage symptoms.

Benefits

- Few to no side effects
- Can improve overall wellbeing
- Can be combined with other treatments
- May reduce stress and pain

Risks/Limitations

- Limited scientific evidence for some
- May be costly (not always covered)
- Requires time and commitment
- May not address severe symptoms

What Matters Most to You?

Check the factors that are most important in your decision:

Maintaining or improving fertility

Reducing or eliminating pain

Avoiding surgery or invasive procedures

Finding a long-term or permanent solution

Minimizing side effects

Improving overall quality of life

Managing costs and insurance coverage

Questions to Ask Your Doctor

- What stage of endometriosis do I have?
- Which treatment do you recommend for my specific situation and why?
- How will this treatment affect my fertility?
- What are the success rates for pain relief with each option?
- What are the possible side effects or complications?
- How long will it take to see results?
- What happens if this treatment doesn't work?
- Will I need ongoing treatment or monitoring?
- Are there clinical trials or newer treatments I should consider?
- Can you refer me to a specialist or support group?

Next Steps

1. Reflect on what matters most to you and your treatment goals.

2. Discuss your options with your healthcare provider, using the questions above.

3. Consider getting a second opinion, especially for surgical options.

4. Connect with support groups or other individuals with endometriosis.

5. Decide on a treatment plan that aligns with your values and goals.

Important: This decision aid is for educational purposes only and is not a substitute for professional medical advice, diagnosis, or treatment. Always consult with your healthcare provider about your specific situation and treatment options.

This decision aid is designed to help you have informed discussions with your healthcare team.

© 2025 Patient Decision Aid Resource

# Microsoft Copilot

**🩺 Patient Decision Aid: Managing Endometriosis**

**🎯 Purpose**

To help individuals with endometriosis understand their treatment options, weigh the benefits and risks, and make decisions aligned with their values and lifestyle.

**📘 Understanding Endometriosis**

- **Definition**: A chronic condition where tissue similar to the uterine lining grows outside the uterus, causing pain, infertility, and other symptoms.
- **Common Symptoms**:
  - Pelvic pain
  - Painful periods
  - Pain during intercourse
  - Fatigue
  - Digestive issues

**🧭 Treatment Options**

| **Option** | **Description** | **Benefits** | **Risks/Considerations** |
| --- | --- | --- | --- |
| **Hormonal Therapy** | Birth control pills, GnRH agonists, progestins | Reduces pain and slows growth | Side effects like mood changes, bone loss |
| **Laparoscopic Surgery** | Minimally invasive removal of endometrial tissue | Symptom relief, fertility improvement | May need repeat surgeries |
| **Hysterectomy & Oophorectomy** | Removal of uterus and/or ovaries | May eliminate symptoms | Irreversible, impacts fertility and hormones |
| **Pain Management** | NSAIDs, physical therapy, acupuncture | Symptom relief | Doesn’t treat root cause |
| **Lifestyle Changes** | Diet, exercise, stress reduction | Improves overall well-being | Requires long-term commitment |

Sources:

**🧠 Decision Factors to Consider**

- **Symptom severity**
- **Desire for fertility**
- **Tolerance for side effects**
- **Preference for non-invasive vs. surgical options**
- **Impact on daily life and mental health**

**🗣️ Questions to Ask Your Provider**

- What are the pros and cons of each treatment?
- How will this affect my fertility?
- What are the long-term outcomes?
- Are there alternatives I haven’t considered?
- How can I monitor my symptoms over time?

**🧩 Values Clarification Exercise**

Rate how important each factor is to you (1 = Not Important, 5 = Very Important):

- Avoiding surgery: ☐1 ☐2 ☐3 ☐4 ☐5
- Preserving fertility: ☐1 ☐2 ☐3 ☐4 ☐5
- Minimizing side effects: ☐1 ☐2 ☐3 ☐4 ☐5
- Quick symptom relief: ☐1 ☐2 ☐3 ☐4 ☐5
- Long-term management: ☐1 ☐2 ☐3 ☐4 ☐5

# Google Gemini

Patient Decision Aid: Managing Endometriosis

**Introduction**

**Endometriosis** is a chronic condition where tissue similar to the lining of the uterus (endometrium) grows outside the uterus. This can cause chronic pain, severe menstrual cramps, pain during sex, and can affect fertility.

This decision aid is designed to help you, in consultation with your healthcare provider, understand your treatment options and choose the path that best aligns with your values, goals, and lifestyle.

**Your current decision**: Which treatment option is best for managing my endometriosis symptoms?

**Disclaimer**: *This information is not a substitute for professional medical advice, diagnosis, or treatment. Always seek the advice of your physician or other qualified health provider with any questions you may have regarding a medical condition.*

**Step 1: Clarify Your Goals and Concerns**

Reflect on and discuss these key areas with your doctor:

| Key Goal | Option 1: Pain Relief | Option 2: Fertility Preservation | Option 3: Long-Term Management |
| --- | --- | --- | --- |
| **Priority Level (Low, Medium, High)** |  |  |  |
| Concerns | *e.g., Side effects, need for surgery, cost* |  |  |

| Question | Your Answer |
| --- | --- |
| **How much does the pain impact my daily life?** (Scale of 1-10) |  |
| **Am I actively trying to conceive or planning to in the near future?** | (Yes/No) |
| **How willing am I to manage potential side effects from medications?** (Very/Somewhat/Not at all) |  |

**Step 2: Understand Your Treatment Options**

Treatment for endometriosis generally focuses on pain management, slowing the progression of the disease, and, for some, improving fertility. The main categories are **Medication** and **Surgery**.

**A. Medication (Non-Surgical Management)**

| Treatment | How it Works | Pros (Advantages) | Cons (Disadvantages) |
| --- | --- | --- | --- |
| **Pain Relievers** (NSAIDs like ibuprofen) | Reduces inflammation and mild pain. | **Widely available**, generally few side effects, inexpensive. | Doesn't treat the endometriosis; only masks pain; not effective for severe pain. |
| **Hormonal Contraceptives** (Pills, Patch, Ring, IUD) | Suppresses the menstrual cycle, reducing endometrial growth and bleeding. | Effective for **pain control; reversible**; non-invasive; also provides contraception. | Possible side effects: **mood changes, irregular bleeding, weight gain**. *Cannot be used if trying to conceive.* |
| **Progestins** (Oral, injection, implant) | Suppresses the menstrual cycle and estrogen production. | **Highly effective for pain**; may improve symptoms when contraceptives fail. | Can cause side effects like **unpredictable bleeding, mood changes, decreased bone density** with long-term use. *Cannot be used if trying to conceive.* |
| GnRH Agonists/Antagonists (Injection/Pill) | Induces a temporary, reversible, menopause-like state to stop the cycle entirely. | **Very strong pain relief**; most effective for severe symptoms. | Causes **menopause-like side effects** (hot flashes, bone density loss); generally used for **short periods** (e.g., 6 months); **High cost**. *Cannot be used if trying to conceive.* |

**B. Surgical Management**

| Treatment | How it Works | Pros (Advantages) | Cons (Disadvantages) |
| --- | --- | --- | --- |
| Conservative Surgery (Laparoscopy with Excision or Ablation) | Surgically removes (excision) or destroys (ablation) the endometrial implants. | Reduces pain; may improve fertility; is diagnostic and therapeutic. | Implants may return (recurrence); requires general anesthesia; risk of surgical complications. |
| Definitive Surgery (Hysterectomy and removal of ovaries/fallopian tubes) | Removes the uterus (hysterectomy) and usually the ovaries (oophorectomy) to eliminate all hormone-producing organs. | Eliminates the source of the problem; often the most permanent solution for pain. | Infertility (cannot conceive); requires major surgery and recovery; causes immediate menopause if ovaries are removed, requiring hormone replacement therapy (HRT). |

**Step 3: Weighing the Trade-offs**

Use this table to compare the options based on your personal priorities.

| Feature | Hormonal Contraceptives | GnRH Agonists | Conservative Surgery | Hysterectomy |
| --- | --- | --- | --- | --- |
| **Pain Relief Effectiveness** | Moderate to High | High | High (Initially) | Very High |
| **Effect on Fertility** | Negative (while taking) | Negative (while taking) | May Improve | **Permanent loss** |
| **Invasiveness** | Low (Oral/IUD) | Medium (Injection) | High (Surgery) | Very High (Major Surgery) |
| **Risk of Recurrence** | High (if stopped) | High (if stopped) | Moderate to High | Low (especially with ovary removal) |
| **Time to Recovery** | Minimal | Minimal | Days to Weeks | Weeks to Months |

Step 4: Make a Decision

Based on the information above and your conversations with your healthcare provider, discuss the best approach for you.

| Scenario | Recommended Primary Approach | Why? |
| --- | --- | --- |
| Mild Pain; Not Trying to Conceive | Pain Relievers and/or Hormonal Contraceptives. | Low invasiveness, good symptom control. |
| Severe Pain; Not Trying to Conceive | Hormonal Contraceptives, Progestins, or GnRH Agonists. | Strongest non-surgical pain relief; avoids surgery. |
| Focus is on Fertility and Pain | Conservative Surgery (Excision) followed by fertility treatments or pain medication. | Surgery can remove implants, which may improve conception rates. |
| Severe, Recurring Pain; Completed Childbearing | Definitive Surgery (Hysterectomy with Oophorectomy). | Offers the most permanent relief from pain. |

My Decision:

I plan to pursue [Insert Chosen Treatment].

My Next Step:

I will discuss [Specific questions/concerns] with my doctor at my next appointment.
