# Supplementary material for: Digital Patient Decision Aids for Endometriosis Management: A Scoping Review
Source: Health Expect. 2026 Sep 25;29(5):e70881. doi: 10.1111/hex.70881 (PMC13614076; doi:10.1111/hex.70881)
Supplement: Supplementary file 2 [file HEX-29-e70881-s002.docx]

# Appendix 4

| **Category** | **Description / Definition** |
| --- | --- |
| **Administrative & Study Info** | |
| DOI or link to the resource | DOI number or link. |
| First Author, Year | Primary bibliographic information used to identify the source. |
| Country | Geographic origin of the study or the location where the PtDA was developed. |
| Aim | The stated objective of the research or the primary goal of the decision aid tool. |
| Study Type / Source | The nature of the source material (e.g., peer-reviewed article, conference abstract, website, or PDF). |
| Origin | Whether the publishing organization is a public institution (government, university) or a private entity (commercial company, private foundation). |
| **Population & Setting** | |
| Population | Description of the target users (e.g., patients with endometriosis, healthcare professionals). |
| Sample Size | The total number of participants or included in the study or evaluation. |
| Age | Age extracted as a range, a mean or a median. |
| Gender | Gender of the participants or intended users. |
| Setting | The clinical or social context where the PtDA is intended to be used (e.g., hospital, home, or online). |
| **PtDA Definition & Digital Context** | |
| Concept: PtDA Definition | Assessment of whether the tool meets the three core criteria: (1) states the decision, (2) provides evidence-based info, and (3) helps clarify patient values. |
| Concept: Digital | Assessment of whether the tool is digital. |
| **Methodology and Key Findings** | |
| Development Process | Description of how the tool was created (e.g., co-creation with patients, expert panels). |
| Evaluation Methods | The metrics used to test the tool, such as user experience. |
| Patient involvement | Describe whether patients were involved and, if so, how they were included (e.g., in a steering group) |
| Outcomes | Variables, endpoints, or phenomena measured. |
| Results | Data, findings, or statistical outputs produced during the study. |
| **IPDAS criteria** | |
| Description of the health condition or problem | The patient decision aid describes the health condition or problem (treatment, procedure, or investigation) for which the index decision is required. |
| Statement of the decision to be considered | The patient decision aid explicitly states the decision that needs to be considered (index decision). |
| Description of the available options | The patient decision aid describes the options available for the index decision. |
| Description of the positive features (benefits/advantages) of each option | The patient decision aid describes the positive features (benefits or advantages) of each option. |
| Description of the negative features (harms/side effects/disadvantages) of each option | The patient decision aid describes the negative features (harms, side effects, or disadvantages) of each option. |
| Description of what it is like to experience the consequences of the options | The patient decision aid describes what it is like to experience the consequences of the options (e.g., physical, psychological, social). |
| Balanced presentation of positive and negative features | The patient decision aid shows the negative and positive features of options with equal detail (e.g., using similar fonts, sequence, presentation of statistical information). |
| Citation of the evidence used | The patient decision aid (or associated documentation) provides citations to the evidence selected. |
| Availability of production/publication date | The patient decision aid (or associated documentation) provides a production or publication date. |
| Information on update policy | The patient decision aid (or associated documentation) provides information about the update policy. |
| Information on uncertainty in probabilities | The patient decision aid provides information about the levels of uncertainty around event or outcome probabilities (e.g., by giving a range or by using phases such as “our best estimate is . . .”). |
| Information on funding source for development | The patient decision aid (or associated documentation) provides information about the funding source used for development. |
| **TiDIER Items** | |
| Name | Provide the name or a phrase that describes the intervention. |
| WHY | Describe any rationale, theory, or goal of the elements essential to the intervention. |
| WHAT : materials | Describe any physical or informational materials used in the intervention, including those provided to participants or used in intervention delivery or in training of intervention providers. Provide information on where the materials can be accessed (e.g. online appendix, URL). |
| WHAT: procedures | Describe each of the procedures, activities, and/or processes used in the intervention, including any enabling or support activities. |
| HOW: mode of delivery | Describe the modes of delivery (e.g. face-to-face or by some other mechanism, such as internet or telephone) of the intervention and whether it was provided individually or in a group. |
| WHEN and HOW MUCH | Describe the number of times the intervention was delivered and over what period of time including the number of sessions, their schedule, and their duration, intensity or dose. |
| TAILORING | If the intervention was planned to be personalised, titrated or adapted, then describe what, why, when, and how. |
| MODIFICATIONS | If the intervention was modified during the course of the study, describe the changes (what, why, when, and how). |
| WHO provided | For each category of intervention provider (e.g. psychologist, nursing assistant), describe their expertise, background and any specific training given. |
| HOW WELL: planned | If intervention adherence or fidelity was assessed, describe how and by whom, and if any strategies were used to maintain or improve fidelity, describe them. |
| HOW WELL: actual | If intervention adherence or fidelity was assessed, describe the extent to which the intervention was delivered as planned. |
| **Additional considerations** | |
| Linguistic Voice | Refers to the grammatical person and linguistic framing used in the tool's text (e.g., first-person "I/me," second-person "you," or third-person "the patient"). |
| SDM & Professionals | How the tool addresses Shared Decision-Making and the role of healthcare providers in the process. |
| Complementary Medicine | Whether the tool includes non-pharmacological or alternative therapies (e.g., diet, exercise, or psychological support). |
