# Supplementary material for: Digital Patient Decision Aids for Endometriosis Management: A Scoping Review
Source: Health Expect. 2026 Sep 25;29(5):e70881. doi: 10.1111/hex.70881 (PMC13614076; doi:10.1111/hex.70881)
Supplement: Supplementary file 3 [file HEX-29-e70881-s003.docx]

| **Criteria** | **IPDAS definition** | **Minimum requirements defined by the research team** |
| --- | --- | --- |
| Qualifying criteria | The patient decision aid describes the health condition or problem (treatment, procedure, or investigation) for which the index decision is required. | Include at least one sentence clearly describing the health condition or problem, as well as the relevant treatment, procedure, or investigation. |
| Qualifying criteria | The patient decision aid explicitly states the decision that needs to be considered (index decision). | Include at least one sentence explicitly stating the decision that needs to be considered by the patient. |
| Qualifying criteria | The patient decision aid describes the options available for the index decision. | Provide a clear description of each available option. |
| Qualifying criteria | The patient decision aid describes the positive features (benefits or advantages) of each option. | Provide a description of the benefits (positive features) of each option. |
| Qualifying criteria | The patient decision aid describes the negative features (harms, side effects, or disadvantages) of each option. | Provide a description of the risks, limitations, or drawbacks (negative features) of each option. |
| Qualifying criteria | The patient decision aid describes what it is like to experience the consequences of the options (e.g., physical, psychological, social). | Descriptions should incorporate supportive elements such as personal stories, example scenarios, FAQs, or other illustrative content to enhance understanding. A general clinical statement on its own is not sufficient. For instance, the following description is too limited: “This procedure can relieve pain in mild to moderate endometriosis and may increase the likelihood of becoming pregnant.” |
| Certification criteria | The patient decision aid shows the negative and positive features of options with equal detail (e.g., using similar fonts, sequence, presentation of statistical information). | For each option, include both positive and negative features in a balanced way. |
| Certification criteria | The patient decision aid (or associated documentation) provides citations to the evidence selected. | Include clear references or links to the external sources used to develop the material. Simply stating “developed by the clinical team” is not sufficient. Even if the search method is described, failing to specify the exact sources used for the PtDA remains insufficient. |
| Certification criteria | The patient decision aid (or associated documentation) provides a production or publication date. | Include the production date or the date of the most recent update. |
| Certification criteria | The patient decision aid (or associated documentation) provides information about the update policy. | Specify the date of the next planned update and explicitly describe the process that will be used to update the document. |
| Certification criteria | The patient decision aid provides information about the levels of uncertainty around event or outcome probabilities (e.g., by giving a range or by using phases such as “our best estimate is . . .”). | Include numerical values or ranges to communicate uncertainty when relevant. Qualitative expressions alone (such as “may” or “might”) are not sufficient. |
| Certification criteria | The patient decision aid (or associated documentation) provides information about the funding source used for development. | Explicitly state the name of the funding source. |
| NB: When the tool provided a link to a website, only the information available on the specific webpage linked by the tool was taken into account. | | |

Table S2: Researcher-Defined Criteria for Assessing IPDAS Compliance
