# Supplementary material for: Digital Patient Decision Aids for Endometriosis Management: A Scoping Review
Source: Health Expect. 2026 Sep 25;29(5):e70881. doi: 10.1111/hex.70881 (PMC13614076; doi:10.1111/hex.70881)
Supplement: Supplementary file 4 [file HEX-29-e70881-s004.docx]

# Appendix 2

A comprehensive search strategy was developed and executed for each database.To ensure transparency, peer review, and reproducibility, the full search strategies, including all keywords and MeSH terms, Boolean operators, truncation, planned limits (e.g., language and publication date), and the number of records retrieved, are presented below:

**Database search strategies**

### Medline Ovid ALL Database coverage: 1946 to July 22, 2025 358 results on July 23, 2025

(Endometriosis/ OR endometrioma*.ti,ab,kf. OR endometrios*.ti,ab,kf.)

**AND** **(**Decision Support Techniques/ OR ((decision* OR decid*) ADJ4 (aid* OR algorithm* OR box* OR instrument OR intervention* OR model* OR process* OR material* OR method* OR program* OR support* OR system* OR technique* OR technolog* OR tool*)).ti,ab,kf. OR (interactive health communication*).ti,ab,kf. OR (interactive ADJ (internet OR online OR graphic* OR booklet*)).ti,ab,kf.

OR

**(**(Decision Making/ OR Decision Making, Shared/ OR Patient Participation/ OR Patient Preference/ OR exp Patients/px OR Patient Satisfaction/ OR exp Health Education/ OR exp Health Education/ OR Educational Technology/ OR Informed Consent/ OR Choice Behavior/ OR (**decision*** OR choice behavio?r OR (patient* ADJ3 (choice* OR engagement OR experience OR expectation* OR involvement OR participat* OR preferenc* OR satisfaction))).ti,ab,kf. OR (informed consent OR risk communication OR risk assessment OR risk information).ti,ab,kf.) **AND** (Algorithms/ OR exp Artificial Intelligence/ OR exp Internet*/ OR Internet-Based Intervention/* OR exp Medical Informatics/ *OR Information Systems/* OR exp Software/ *OR Mobile Applications/* OR ("AI" OR algorithm* OR apps OR "artificial intelligence" OR chatbot* OR "clinical decision support*" OR "computational intelligence*" OR computer* OR digital OR digitis* OR digitiz* OR ehealth OR e-health OR electronic* OR informatics OR "information system*" OR interactive OR internet OR "machine intelligence*" OR "machine learning" OR mobile OR online OR platform* OR "portable electronic app*" OR "smartphone app*" OR software* OR web).ti,ab,kf.)**)**)

### Embase.com 867 results on July 23, 2025

('endometriosis'/exp OR (endometrioma* OR endometrios*):ti,ab,kw)

**AND** ('decision support system'/de OR ((decision* OR decid*) NEAR/4 (aid* OR algorithm* OR box* OR instrument OR intervention* OR model* OR process* OR material* OR method* OR program* OR support* OR system* OR technique* OR technolog* OR tool*)):ti,ab,kw OR (interactive health communication*):ti,ab,kw OR (interactive NEXT/1 (internet OR online OR graphic* OR booklet*)):ti,ab,kw

OR

(('decision making'/exp OR 'patient attitude'/de OR 'patient engagement'/de OR 'patient expectation'/de OR 'patient experience'/de OR 'patient participation'/de OR 'patient preference'/de OR 'patient satisfaction'/de OR 'health education'/exp OR 'educational technology'/de OR 'informed consent'/exp OR (decision* OR "choice behavio$r" OR (patient* NEAR/3 (choice* OR engagement OR experience OR expectation* OR involvement OR participat***** OR preferenc***** OR satisfaction))):ti,ab,kw OR ("informed consent" OR "risk communication" OR "risk assessment" OR "risk information"):ti,ab,kw) **AND** ('algorithm'/de OR 'artificial intelligence'/exp OR 'machine learning'/exp OR 'Internet'/de OR 'web-based intervention'/de OR 'medical informatics'/exp OR 'information system'/exp OR 'mobile application'/exp OR ("AI" OR algorithm* OR apps OR "artificial intelligence" OR chatbot* OR "clinical decision support*" OR "computational intelligence*" OR computer* OR demateriali* OR digital OR digitis* OR digitiz* OR ehealth OR e-health OR electronic* OR "information system*" OR informatics OR interactive OR internet OR "machine intelligence*" OR "machine learning" OR mobile OR online OR platform* OR "portable electronic app*" OR "smartphone app*" OR software* OR web):ti,ab,kw)**)**)

### CINAHL EBSCO 115 results on July 23, 2025

(MH "Endometriosis" OR XB (endometrioma* OR endometrios*))

**AND** (MH "Decision Support Techniques" OR XB (((decision* OR "choice behavio#r" OR decid*) N3 (aid* OR algorithm* OR box* OR instrument OR intervention* OR model* OR process* OR material* OR method* OR program* OR support* OR system* OR technique* OR technolog* OR tool*)) OR "interactive health communication*" OR (interactive N1 (internet OR online OR graphic* OR booklet*)))

OR

((MH "Decision Making" OR MH "Patient Participation+" OR MH "Decision Making, Patient" *OR MH "Decision Making, Shared" OR MH "Patient Satisfaction+"* OR MH "Patient Preference"

OR MH "Health Education" OR MH "Educational Technology" OR MH "Consent" OR XB decision* OR XB (patient* N2 (choice* OR engagement OR experience OR expectation* OR involvement OR participat* OR preferenc* OR satisfaction)) OR XB ("informed consent" OR "risk communication" OR "risk assessment" OR "risk information")) **AND** (MH "Algorithms" OR MH "Artificial Intelligence+" OR MH "Internet+" OR MH "Internet-Based Intervention" OR MH "Medical Informatics+" OR MH "Information Systems" OR MH "Software+" OR MH "Mobile Applications" OR XB ("AI" OR algorithm* OR apps OR "artificial intelligence" OR chatbot* OR "clinical decision support*" OR "computational intelligence*" OR computer* OR demateriali* OR digital OR digitis* OR digitiz* OR ehealth OR "e-health" OR electronic* OR informatics OR "information system*" OR interactive OR internet OR "machine intelligence*" OR "machine learning" OR mobile OR online OR platform* OR "portable electronic app*" OR "smartphone app*" OR software* OR web))))

### Cochrane Central Register of Controlled Trials Wiley Database coverage: Issue 6 of 12, June 2025 91 results on July 23, 2025

(endometrioma* OR endometrios*):ti,ab,kw

AND

(((decision* OR decid*) NEAR/4 (aid* OR algorithm* OR box* OR instrument OR intervention* OR model* OR process* OR material* OR method* OR program* OR support* OR system* OR technique* OR technolog* OR tool*)):ti,ab,kw OR ("interactive health" NEXT communication*):ti,ab,kw OR (interactive NEAR/1 (internet OR online OR graphic* OR booklet*)):ti,ab,kw

OR

((decision* OR (patient* NEAR/3 (choice* OR engagement OR experience OR expectation* OR involvement OR participat* OR preferenc* OR satisfaction)) OR ("health education" OR "educational technology" OR "informed consent" OR "choice behavior" OR "choice behaviour" OR "risk communication" OR "risk assessment" OR "risk information")):ti,ab,kw AND ("AI" OR algorithm* OR apps OR "artificial intelligence" OR chatbot* OR ("clinical decision" NEXT support*) OR ("computational" NEXT intelligence*) OR computer* OR demateriali* OR digital OR digitis* OR digitiz* OR ehealth OR e-health OR electronic* OR informatics OR ("information" NEXT system*) OR interactive OR internet OR ("machine" NEXT intelligence*) OR "machine learning" OR mobile OR online OR platform* OR ("portable electronic" NEXT app*) OR ("smartphone" NEXT app*) OR software* OR web):ti,ab,kw))

### Cochrane Database of Systematic Reviews Wiley Database coverage: Issue 6 of 12, June 2025 5 results on July 23, 2025

(endometrioma* OR endometrios*):ti,ab,kw

AND

(((decision* OR decid*) NEAR/4 (aid* OR algorithm* OR box* OR instrument OR intervention* OR model* OR process* OR material* OR method* OR program* OR support* OR system* OR technique* OR technolog* OR tool*)):ti,ab,kw OR ("interactive health" NEXT communication*):ti,ab,kw OR (interactive NEAR/1 (internet OR online OR graphic* OR booklet*)):ti,ab,kw

OR

((decision* OR (patient* NEAR/3 (choice* OR engagement OR experience OR expectation* OR involvement OR participat* OR preferenc* OR satisfaction)) OR ("health education" OR "educational technology" OR "informed consent" OR "choice behavior" OR "choice behaviour" OR "risk communication" OR "risk assessment" OR "risk information")):ti,ab,kw AND ("AI" OR algorithm* OR apps OR "artificial intelligence" OR chatbot* OR ("clinical decision" NEXT support*) OR ("computational" NEXT intelligence*) OR computer* OR demateriali* OR digital OR digitis* OR digitiz* OR ehealth OR e-health OR electronic* OR informatics OR ("information" NEXT system*) OR interactive OR internet OR ("machine" NEXT intelligence*) OR "machine learning" OR mobile OR online OR platform* OR ("portable electronic" NEXT app*) OR ("smartphone" NEXT app*) OR software* OR web):ti,ab,kw))

### Web of Science – Core collection

### Science Citation Index Expanded (1900-present), Social Sciences Citation Index (1900-present), Arts & Humanities Citation Index (1975-present), Conference Proceedings Citation Index - Science (1990-present), Conference Proceedings Citation Index – Social Science & Humanities (1990-present), Book Citation Index - Science (2005-present), Book Citation Index – Social Sciences & Humanities (2005-present), Emerging Sources Citation Index (last 5 years), Current Chemical Reactions (1985-present), Index Chemicus (1993-present) 363 results on July 23, 2025

TS=(endometrioma* OR endometrios*)

**AND** (TS=(((decision* OR decid*) NEAR/3 (aid* OR algorithm* OR box* OR instrument OR intervention* OR model* OR process* OR material* OR method* OR program* OR support* OR system* OR technique* OR technolog* OR tool*)) OR "interactive health communication*" OR (interactive NEAR/1 (internet OR online OR graphic* OR booklet*)))

OR

(TS=(decision* OR (patient* NEAR/2 (choice* OR engagement OR experience OR expectation* OR involvement OR participat* OR preferenc* OR satisfaction)) OR ("health education" OR "educational technology" OR "informed consent" OR "choice behavior" OR "choice behaviour" OR "risk communication" OR "risk assessment" OR "risk information")) **AND** TS=("AI" OR algorithm* OR apps OR "artificial intelligence" OR chatbot* OR "clinical decision support*" OR "computational intelligence*" OR computer* OR demateriali* OR digital OR digitis* OR digitiz* OR ehealth OR e-health OR electronic* OR informatics OR "information system*" OR interactive OR internet OR "machine intelligence*" OR "machine learning" OR mobile OR online OR platform* OR "portable electronic app*" OR "smartphone app*" OR software* OR web)))

**Additional searches**

### ProQuest Dissertations & Theses A&I

### (2000–2029) - Doctoral dissertations only 21 results on July 23, 2025

(endometrios* OR endometrioma*) AND ("patient decision aid*") AND ("decision support" OR digital OR online OR web)

### BASE (Bielefeld Academic Search Engine)

Search [all fields]
13 results on July 23, 2025

(endometrios* OR endometrioma*) AND (decision*) AND ("decision support" OR digital OR online OR web)

### Emerald Insight

53 results on July 23, 2025

(endometrios* OR endometriom*) AND decision* AND ("decision support" OR digital OR online OR web)

### Trip Database Pro

5 results on July 23, 2025

(endometrios* OR endometrioma*) AND (decision*) AND ("decision support" OR digital OR online OR web)

### Google Scholar

Endometriosis "patient decision aid"|"patient decision aids"|"shared decision making" digital|online|web

intitle:endometriosis "patient decision aid"|"shared decision making" digital|online

intitle:"patient decision aid" endometriosis digital|online

intitle:"shared decision making" endometriosis digital|online

*The search was conducted in July 22, 2025 and yielded 5670 references. A shared database of 215 references was compiled based on the above strategies, using the Publish or Perish tool, version 8 (Harzing.com)*
