# Supplementary material for: Digital Patient Decision Aids for Endometriosis Management: A Scoping Review
Source: Health Expect. 2026 Sep 25;29(5):e70881. doi: 10.1111/hex.70881 (PMC13614076; doi:10.1111/hex.70881)
Supplement: Supplementary file 5 [file HEX-29-e70881-s005.docx]

# Appendix 5

| **Administrative & Study Info** | | | | | |
| --- | --- | --- | --- | --- | --- |
| **DOI or link to the ressource** | **First Author, Year** | **Country** | **Aim** | **Study Type** | **Origin** |
| <https://doi.org/10.1136/bmjebm-2024-SDC.210>  https://decisionscompartides.gencat.cat/es/decidir-sobre/endometriosi/ | Ramos-Masdeu (2024) | Spain | Co-creation and evaluation of a Shared Decision-Making Tool for professionals and patients with endometriosis. | Abstract | Public |
| <https://www.cham.org/HealthwiseArticle.aspx?id=tv7242>. | Healthwise (2025) | USA | Support shared decision-making regarding the choice of hysterectomy and oophorectomy for endometriosis symptoms. | Website | Private |
| https://www.keuzehulp-vruchtbaarheidsbehoud-endometriose.nl/ | VUmc & AMC (2018) | Netherland | Facilitate medical conversations and decision-making specifically regarding fertility preservation in the context of endometriosis. | Website | Public |
| https://www.informedhealth.org/pdf/endometriose/da_treatments_endometriosis.pdf | IQWiG (2024) | Germany | Assist patients in choosing a suitable treatment (medication or surgery) in collaboration with their healthcare providers. | Online PDF | Public |
| https://www.stgeorges.nhs.uk/wp-content/uploads/2024/03/GYN_MOE.pdf | NHS Trust (2024) | United Kingdom | Provide evidence-based information on endometriosis management options, including a balanced overview of benefits and risks. | Online PDF | Public |
| **Link to the resource** | **Platform name, Underlying model, Year** | **Country** | **Aim** | **Study Type** | **Origin** |
| <https://chatgpt.com/> | OpenAI ChatGPT (GPT-4o) (2025) | USA | Help individuals understand endometriosis, explore treatment options, and prepare for collaborative healthcare consultations. | LLM Output | Private |
| <https://www.perplexity.ai/> | Perplexity AI (standard non-premium configuration) (2025) | USA | Empower self-advocacy and shared decision-making by providing balanced info on treatments and quality-of-life impacts. | LLM Output | Private |
| <https://claude.ai> | Anthropic Claude (Claude Sonnet 4.5) (2025) | USA | Support informed choices about medical and surgical treatment options in coordination with a healthcare team. | LLM Output | Private |
| <https://copilot.microsoft.com/> | Microsoft Copilot (standard non-premium configuration) (2025) | USA | Enable individuals to weigh treatment benefits and risks to make decisions aligned with personal values and lifestyle. | LLM Output | Private |
| https://gemini.google.com/ | Google Gemini (Gemini 2.5 Flash) (2025) | USA | Assist users in choosing a treatment path that aligns with personal goals and values through consultation with a provider. | LLM Output | Private |

| **Population & Setting** | | | | | |
| --- | --- | --- | --- | --- | --- |
|  | Population | Sample size | Age | Gender | Setting |
| Ramos-Masdeu (2024) | Patients with endometriosis. | 12,462 visitors of the website | NR | Inclusive (people who have a uterus, including both cisgender women and people with other gender identities) | NR |
| Healthwise (2025) | Intended for professional evaluation and not intended for distribution directly to patients or consumers.  Targeted patients: women who have tried hormone therapy and have had laparoscopic surgery to remove tissue but still have severe symptoms. | NR | NR | Do not specify a gender "For individuals with endometriosis" | NR |
| VUmc & AMC (2018) | Patients with endometriosis. | NR | NR | Do not specify a gender "For individuals with endometriosis" | NR |
| IQWiG (2024) | Patients with endometriosis. | NR | NR | Do not specify a gender "For individuals with endometriosis" | NR |
| NHS Trust (2024) | Patients with endometriosis. | NR | NR | Do not specify a gender "For individuals with endometriosis" | NR |
| OpenAI ChatGPT (2025) | Patients with endometriosis. | N/A | N/A | Do not specify a gender "For individuals with endometriosis" | NR |
| Perplexity AI (2025) | Patients with endometriosis. | N/A | N/A | Do not specify a gender "For individuals with endometriosis" | NR |
| Anthropic Claude (2025) | Patients with endometriosis. | N/A | N/A | Do not specify a gender "For individuals with endometriosis" | NR |
| Microsoft Copilot (2025) | Patients with endometriosis. | N/A | N/A | Do not specify a gender "For individuals with endometriosis" | NR |
| Google Gemini (2025) | Patients with endometriosis. | N/A | N/A | Do not specify a gender "For individuals with endometriosis" | NR |

| **PtDA Definition & Digital Context** | | |
| --- | --- | --- |
|  | Concept: PtDA Definition | Concept: Digital |
| Ramos-Masdeu (2024) | Yes (All 3 criteria) \| | Yes digital |
| Healthwise (2025) | Yes (All 3 criteria) \| | Yes digital |
| VUmc & AMC (2018) | Yes (All 3 criteria) \| | Yes digital |
| IQWiG (2024) | Yes (All 3 criteria) \| | Yes digital |
| NHS Trust (2024) | Yes (All 3 criteria) \| | Yes digital |
| OpenAI ChatGPT (2025) | Yes (All 3 criteria) \| | Yes digital |
| Perplexity AI (2025) | Yes (All 3 criteria) \| | Yes digital |
| Anthropic Claude (2025) | Yes (All 3 criteria) \| | Yes digital |
| Microsoft Copilot (2025) | Yes (All 3 criteria) \| | Yes digital |
| Google Gemini (2025) | Yes (All 3 criteria) \| | Yes digital |

| **Methodology and Key Findings** | | | | | |
| --- | --- | --- | --- | --- | --- |
|  | Development Process | Evaluation Methods | Patient involvement | Outcomes | Results |
| Ramos-Masdeu (2024) | The co-creation process engaged 9 endometriosis experts, 2 shared decision-making experts, 1 patient experience expert, 11 patients and 2 patient associations | The number of web visits were followed-up and user experience was analyzed through a questionnaire. | Yes, involved 1 patient experience expert, 11 patients and 2 patient associations. | Number of visits,  user experience and satisfaction score. | Within five months of publication, it garnered 12,462 users, with a satisfaction rate averaging 3.4 out of 5 and 30% of users assigning the highest score. |
| Healthwise (2025) | Digital health education content is developed and sourced using current guidelines from government agencies, expert medical and health care organizations. The clinical education content is developed by a multidisciplinary team that includes clinicians, writers, editors, and independent contractors. The development of content involves a minimum of two clinical reviewers, at least one of whom is a specialist in the area under consideration. This ensures that each piece of content is not only accurate, but is also relevant to the latest medical guidelines. Furthermore, each reviewer’s credentials are verified at regular intervals—typically every one to two years—to confirm they remain in good standing. | NR | NR | NR | NR |
| VUmc & AMC (2018) | The content of this Decision Aid was created during eight thinking sessions with patient experts, medical specialists and a psychologist. | NR | Yes, involved patient experts. | NR | NR |
| IQWiG (2024) | The format of this decision aid is based on the following:  • Ottawa Personal Decision Guide. O’Connor, Stacey, Jacobsen 2012. Ottawa Hospital Research  Institute and University of Ottawa, Canada.  • MAKING SDM A REALITY – Hospital-wide shared decision making – G-BA Innovation fund 2023.  • Institute for Quality and Efficiency in Health Care (IQWiG, Germany). Development of a decision aid  for hysterectomy: Rapid Report; Commission P18-01. 2019 | NR | NR | NR | NR |
| NHS Trust (2024) | NR | NR | NR | NR | NR |
| OpenAI ChatGPT (2025) | NR | N/A | N/A | N/A | N/A |
| Perplexity AI (2025) | NR | N/A | N/A | N/A | N/A |
| Anthropic Claude (2025) | NR | N/A | N/A | N/A | N/A |
| Microsoft Copilot (2025) | NR | N/A | N/A | N/A | N/A |
| Google Gemini (2025) | NR | N/A | N/A | N/A | N/A |

| **IPDAS criteria** | | | | | | | | | | | | |
| --- | --- | --- | --- | --- | --- | --- | --- | --- | --- | --- | --- | --- |
|  | Description of the health condition or problem | Description of the decision to be considered | Description of the available options | Description of the positive features (benefits/advantages) of each option | Description of the negative features (harms/side effects/disadvantages) of each option | Description of what it is like to experience the consequences of the options | Balanced presentation of positive and negative features | Citation of the evidence used | Availability of production/publication date | Information on update policy | Information on uncertainty in probabilities | Information on funding source for development |
| Ramos-Masdeu (2024) | yes | yes | yes | yes | yes | yes | yes | yes | yes | no | yes | no |
| Healthwise (2025) | yes | yes | yes | yes | yes | yes | yes | no | yes | yes | no | yes |
| VUmc & AMC (2018) | yes | yes | yes | yes | yes | no | no | no | yes | no | no | no |
| IQWiG (2024) | yes | yes | yes | yes | yes | no | yes | no | yes | yes | yes | yes |
| NHS Trust (2024) | yes | yes | yes | yes | yes | no | no | no | yes | no | yes | no |
| OpenAI ChatGPT (2025) | yes | yes | yes | yes | yes | no | yes | no | no | no | no | no |
| Perplexity AI (2025) | yes | yes | yes | no | no | no | no | yes | no | no | no | no |
| Anthropic Claude (2025) | yes | yes | yes | yes | yes | no | yes | no | no | no | no | no |
| Microsoft Copilot (2025) | yes | yes | yes | yes | yes | no | yes | no | no | no | no | no |
| Google Gemini (2025) | yes | yes | yes | yes | yes | no | yes | no | no | no | no | no |

| **TiDIER items** | | | | | | | | | | | |
| --- | --- | --- | --- | --- | --- | --- | --- | --- | --- | --- | --- |
|  | Provide the name or a phrase that describes the intervention. | WHY | WHAT : materials | WHAT : procedures | HOW : mode of delivery | WHEN and HOW MUCH | TAILORING | MODIFICATIONS | WHO provided | HOW WELL : planned | HOW WELL : actual |
| Ramos-Masdeu (2024) | Shared decision-making in endometriosis | Help patients with endometriosis make informed health decisions. | PtDA | NR | Digital PtDAs | NR | NR | NR | NR | NR | NR |
| Healthwise (2025) | Endometriosis: Should I Have a Hysterectomy and Oophorectomy? | Help patients with endometriosis make informed health decisions. | PtDA | NR | Digital PtDAs | NR | NR | NR | NR | NR | NR |
| VUmc & AMC (2018) | Decision aid for fertility preservation in endometriosis | Help patients with endometriosis make informed health decisions. | PtDA | NR | Digital PtDAs | NR | NR | NR | NR | NR | NR |
| IQWiG (2024) | Decision Aid Endometriosis: What are your treatment options? | Help patients with endometriosis make informed health decisions. | PtDA | NR | Digital PtDAs | NR | NR | NR | NR | NR | NR |
| NHS Trust (2024) | Management of Endometriosis: Information and Decision Aid | Help patients with endometriosis make informed health decisions. | PtDA | NR | Digital PtDAs | NR | NR | NR | NR | NR | NR |
| OpenAI ChatGPT (2025) | Endometriosis: Patient Decision Aid | Help patients with endometriosis make informed health decisions. | PtDA | NR | Digital PtDAs | NR | NR | NR | NR | NR | NR |
| Perplexity AI (2025) | A patient decision aid for individuals with endometriosis | Help patients with endometriosis make informed health decisions. | PtDA | NR | Digital PtDAs | NR | NR | NR | NR | NR | NR |
| Anthropic Claude (2025) | Endometriosis Patient Decision Aid | Help patients with endometriosis make informed health decisions. | PtDA | NR | Digital PtDAs | NR | NR | NR | NR | NR | NR |
| Microsoft Copilot (2025) | Patient Decision Aid: Managing Endometriosis | Help patients with endometriosis make informed health decisions. | PtDA | NR | Digital PtDAs | NR | NR | NR | NR | NR | NR |
| Google Gemini (2025) | Patient Decision Aid: Managing Endometriosis | Help patients with endometriosis make informed health decisions. | PtDA | NR | Digital PtDAs | NR | NR | NR | NR | NR | NR |

| **Additional considerations** | | | |
| --- | --- | --- | --- |
|  | Linguistic Voice | SDM & Professionals | Complementary Medicine |
| Ramos-Masdeu (2024) | 1st/2nd Pers. | Talk about SDM and health professionals.  Include decision-support features with explicit values clarification, comparison tools and consultation preparation support. | yes |
| Healthwise (2025) | 1st/2nd Pers. | Talk about SDM and health professionals.  Include decision-support features with explicit values clarification, comparison tools and consultation preparation support. | no |
| VUmc & AMC (2018) | 1st/2nd Pers. | Talk about SDM and health professionals. | no |
| IQWiG (2024) | 1st/2nd Pers. | Talk about SDM and health professionals.  Include decision-support features with explicit values clarification, comparison tools and consultation preparation support. | no |
| NHS Trust (2024) | 1st/2nd Pers. | Talk about SDM and health professionals. | no |
| OpenAI ChatGPT (2025) | 1st/2nd Pers. | Talk about SDM and health professionals.  Include decision-support features with explicit values clarification.  Outlined next steps in the decision process. | yes |
| Perplexity AI (2025) | 3rd Pers. | Talk about SDM and health professionals.  Include decision-support features. | yes |
| Anthropic Claude (2025) | 1st/2nd Pers. | Talk about SDM and health professionals.  Include decision-support features with explicit values clarification.  Outlined next steps in the decision process. | yes |
| Microsoft Copilot (2025) | 1st/2nd Pers. | Talk about SDM and health professionals.  Include decision-support features with explicit values clarification. | yes |
| Google Gemini (2025) | 1st/2nd Pers. | Talk about SDM and health professionals.  Include decision-support features with explicit values clarification.  Outlined next steps in the decision process. | no |
