# Supplementary material for: Digital Patient Decision Aids for Endometriosis Management: A Scoping Review
Source: Health Expect. 2026 Sep 25;29(5):e70881. doi: 10.1111/hex.70881 (PMC13614076; doi:10.1111/hex.70881)
Supplement: Supplementary file 7 [file HEX-29-e70881-s007.docx]

**Appendix 5. Excluded records due to inaccessibility or withdrawal of PtDAs**

| Reason for exclusion | Record |
| --- | --- |
| No access to full text or PtDA | Sherman K, Armour M, Lau A, Abbott J, Cooper M, van Niekerk L, Schubert R, Ciccia D, Duckworth T. Empowering people living with endometriosis through EndoChoice, a co-designed decision aid to support informed decision-making for endometriosis management  [online]. Macquarie University. [Accessed 17 June 2026]. Available from: <https://researchers.mq.edu.au/en/projects/empowering-people-living-with-endometriosis-through-endochoice-a-/> |
| No access to full text or PtDA | [Patient Decision Aids Research Group](https://decisionaid.ohri.ca/index.html). Patient Decision Aid Summary page, Hormone treatment for endometriosis symptoms – what are my options?  [online]. Ottawa Hospital Research Institute. [Accessed 17 June 2026]. Available from: <https://decisionaid.ohri.ca/AZsumm.php?ID=1893> |
| No access to full text or PtDA (backward citation) | Richter R, Jansen J, van der Kraan J, Abbaspoor W, Bongaerts I, Pouwels F, et al. How inclusive are patient decision aids for people with limited health literacy? An analysis of understandability criteria and the communication about options and probabilities. Med Decis Making. 2025;45(2):143-155. |
| PtDA withdrawn | [Patient Decision Aids Research Group](https://decisionaid.ohri.ca/index.html). Patient Decision Aid Summary page, Endometriosis: Should I Use Hormone Therapy?  [online]. Ottawa Hospital Research Institute. [Accessed 17 June 2026]. Available from:  <https://decisionaid.ohri.ca/Azsumm.php?ID=1021> |
