# Supplementary material for: Digital Patient Decision Aids for Endometriosis Management: A Scoping Review
Source: Health Expect. 2026 Sep 25;29(5):e70881. doi: 10.1111/hex.70881 (PMC13614076; doi:10.1111/hex.70881)
Supplement: Supplementary file 8 [file HEX-29-e70881-s008.docx]

| **Source** | **Criteria** |
| --- | --- |
| IPDAS (32) | Qualifying criteria: Health condition/problem; Explicit decision; Options; Positive features; Negative features; Consequences experience |
|  | Certification criteria: Balanced detail; Evidence citations; Publication date; Update policy; Outcome uncertainty; Funding source |
| TIDieR (33) | Brief name; Why (rationale, theory or goal); What (Materials and Procedures); Who provided; How (mode of delivery); When and how much (number of times and period of time); Tailoring; Modifications; How well (fidelity/adherence) |

Table S1. IPDAS criteria and TIDieR items used to appraise quality and replicability of included PtDAs.
